# Syntax Score I and II for Predicting Carotid Artery Stenosis in Patients with Multivessel Coronary Artery Disease: A Propensity Score Matching Analysis

**DOI:** 10.21470/1678-9741-2019-0067

**Published:** 2019

**Authors:** Semi Ozturk, Mazlum Sahin

**Affiliations:** 1 Department of Cardiology, Haseki Training and Research Hospital, Istanbul, Turkey.; 2 Department of Cardiovascular Surgery, Haseki Training and Research Hospital, Istanbul, Turkey.

**Keywords:** Coronary Artery Disease, Coronary Artery Bypass, Carotid Stenosis, Percutaneous Coronary Intervention, Risk Factors

## Abstract

**Objective:**

To evaluate the predictive accuracy of SYNTAX score (SS) I and II for detecting significant carotid artery stenosis (CAS) in patients with multivessel coronary artery disease undergoing coronary artery bypass grafting (CABG) surgery.

**Methods:**

The study population consisted of 416 patients. Clinical, demographic, and radiological records were retrospectively reviewed. Characteristics of patients with CAS (n=66) and patients without CAS (n=350) were compared before and after propensity score matching analysis.

**Results:**

Patients with significant CAS were older compared to those without significant CAS [(60 (53-65) *vs*. 63 (59-67); *P*=0.01]. However, atherosclerotic risk factors and SS I were similar between groups. SS II CABG and percutaneous coronary intervention (PCI) were significantly higher in patients with CAS [37.4 (30.9-43.5) *vs*. 33.8 (29.9-38.9); *P*=0.02]. After propensity score matching analysis (66 *vs*. 66), age, SS II PCI and CABG were significantly higher in patients with CAS than those without CAS [37.4 (30.9-43.5) *vs*. 33 (29.3-36.9); *P*=0.03]. Age, SS II PCI and CABG were associated with CAS in logistic regression analysis [OR=1.086, 95% CI (1.032-1.143), *P*<0.001; OR=1.054, 95% CI (1.010-1.101), *P*=0.02; OR=1.078, 95% CI (1.029-1.129), *P*<0.01].

In ROC curve analysis, SS II PCI >33.1 had 68.2% sensitivity and 54.6% specificity [AUC=0.624, *P*=0.01, 95% CI (0.536-0.707)] whereas SS II CABG >26.1 had 81.8% sensitivity and 54.6% specificity [AUC=0.670, *P*<0.01, 95% CI (0.583-0.749)] to predict CAS. Pairwise comparison of ROC curves revealed similar statistical accuracy for prediction of CAS (z statistic: 0.683, *P*=0.49)

**Conclusion:**

SS II is useful to predict asymptomatic CAS in patients with multivessel coronary artery disease.

## INTRODUCTION

Multivessel coronary artery disease is often accompanied by involvement of carotid and lower extremity arteries^[1]^. Association between coronary artery disease and carotid artery stenosis (CAS) is well documented in previous studies^[2]^. Coexisting CAS in patients with multivessel coronary artery disease undergoing coronary bypass grafting (CABG) cause worse outcomes^[3]^. Prediction of CAS in patients undergoing CABG may improve outcomes^[4]^.

SYNTAX score (SS) I and II are recent scores which are used for choosing the treatment modality in patients with multivessel disease. During the last decade, several studies showed a close association between these scores and cardiovascular outcomes^[5-8]^. The aim of our study is to determine the association between SS I-II and significant CAS in patients with multivessel disease undergoing CABG.

### Study Population

The study included patients who were scheduled for CABG due to multivessel disease between January 2015 and January 2017. Patients with a history of carotid artery stenting or surgery and history of previous stroke were excluded. Preoperative routine carotid ultrasound evaluation was performed one week before CABG in our institution. Clinical, demographic and radiologic records were retrospectively reviewed.

### Coronary Angiography and SYNTAX I and II Scores

SS I was calculated for each lesion with >50% diameter stenosis in vessels >1.5 mm in diameter. An experienced interventional cardiologist who is blind to the study performed the calculation. The online version was used for the calculation of SS I and II (www.syntaxscore.com).

### Assessment of Carotid Doppler Ultrasound

Bilateral carotid arteries were evaluated by an experienced radiologist. B-mode and Doppler US modalities were used for evaluation. The maximum percentage of diameter reduction was calculated by B-mode. Significant carotid artery disease was defined as a stenosis >50 and a peak systolic velocities >125 cm/s.

### Statistical Analysis

Statistical analysis was performed with SPSS version 22.0 (IBM Corp. Armonk, NY, USA) and MedCalc bvba version 16 (Seoul, South Korea). Data were presented as median (interquartile range) for quantitative variables and as percentages for categorical variables. The normality of data distribution was tested by Kolmogorov-Smirnov tests. Numerical variables were tested with Mann-Whitney U-test and categorical variables were tested using Fisher’s exact test or chi-square test, whichever was suitable. Continuity correction was used when needed. A *P*-value <0.05 was regarded as significant. Since the study was nonrandomized, a logistic regression model with propensity scores was created in order to balance patient characteristics and perform propensity-matched analysis of the patients with and without CAS. SS II variables were not included in the model. The variables used in this model were: hypertension, diabetes mellitus, smoking, and low-density lipoprotein level. One-to-one nearest-neighbor matching was performed using a caliper width of 0.1. The resulting score-matched pairs were used to re-evaluate the analysis. Univariate analysis was performed to determine predictors of CAS. Receiver-operating characteristic (ROC) curve graphics were used to determine the cutoff values of independent predictors.

## RESULTS

The study population consisted of 416 patients who underwent CABG: 40.4% of the patients presented with acute coronary syndrome and 7.2% of the patients had chronic kidney disease (eGFR <60 ml/min/1.73 m^2^). Significant CAS was detected in 66 patients. Demographic, clinical and laboratory characteristics of patients with significant CAS and those without CAS are presented in Table 1. SS II CABG and PCI were significantly higher in patients with CAS [37.4 (30.9-43.5) *vs*. 33.8 (29.9-38.9); *P*=0.02]. Patients with CAS were older, however, atherosclerotic risk factors were similar compared with patients without CAS.

**Table 1:** Characteristics of patients.

	Before matching			After matching		
	Non-significant CAS	Significant CAS	*P*	Non-significant CAS	Significant CAS	*P*
	n=350	n=66		n=66	n=66	
Age (years)	60 (53-65)	63 (59-67)	0.01	57 (51.5-65)	63 (59-67)	0.03
Sex (male), n (%)	284 (81.1)	50 (75.8)	0.40	54 (81.8)	50 (75.8)	0.52
BMI (kg/m^2^)	26.6 (24.2-29.4)	26.4 (24.2-29.7)	0.93	26.7 (24.2-29.7)	26.4 (24.2-29.7)	0.61
Smoking, n (%)	139 (39.7)	23 (34.8)	0.55	22 (33.3)	23 (34.8)	>0.99
HT, n (%)	200 (42.9)	25 (37.9)	0.54	23 (34.8)	25 (37.9)	0.86
DM, n (%)	131(37.4)	20 (30.3)	0.34	23(34.8)	20(30.3)	0.71
COPD, n (%)	59 (16.9)	10 (15.2)	0.87	11 (16.7)	10 (15.2)	>0.99
PAD, n (%)	40 (11.4)	12 (18.2)	0.19	4 (6.1)	12 (18.2)	0.06
EF (%)	50 (45-60)	50 (40-60)	0.09	50 (43.8-60)	50 (40-60)	0.27
CKD	27 (7.7)	3 (4.5)	0.36	2 (3)	3 (4.5)	0.65
eGFR (mL/min/1.73 m^2^)	92 (78-103)	93 (79.8-104.3)	0.95	96 (74.8-105.3)	93 (79.8-104.3)	0.61
LDL	126 (100-179)	126 (100-175)	0.48	116 (99.5-175)	126 (100-175)	0.59
CRP	7 (4-10)	6 (4-10)	0.60	6.5 (4.8-10)	6 (4-10)	0.90
MI at presentation	141 (40.3)	27 (41.5)	0.85	28 (42.4)	27 (41.5)	0.92
SYNTAX I	19.5 (14-25.5)	19.3 (13-27.1)	0.66	20.5 (14-27.5)	19.3 (13-27.1)	0.47
SYNTAX II PCI	33.8 (29.9-38.9)	37.4 (30.9-43.5)	0.02	33 (29.3-36.9)	37.4 (30.9-43.5)	0.01
SYNTAX II CABG	25 (21.4-31)	29 (26.7-35.2)	<0.01	25.6 (22.8-31.2)	29 (26.7-35.2)	<0.01

BMI=body mass index; CABG=coronary artery bypass grafting; CAS=carotid artery stenosis; CKD=chronic kidney disease; CRP=C-reactive protein; DM=diabetes mellitus; EF=ejection fraction; eGFR=estimated glomerular filtration rate; HT=hypertension ; LDL=low-density lipoprotein; MI=myocardial infarction; PAD=peripheral arterial disease; PCI=percutaneous coronary intervention

After propensity score matching analysis (66 *vs*. 66), SS II PCI and CABG were significantly higher in patients with CAS than those without CAS [37.4 (30.9-43.5) *vs*. 33 (29.3-36.9); *P*=0.03]. SS I was similar between groups. Age was significantly higher in patients with CAS. Characteristics of the population before and after matching are presented in Table 1.

In the matched population, age, SS II PCI and CABG were associated with CAS in univariate logistic regression analysis [OR=1.086, 95% CI (1.032-1.143), *P*<0.001; OR=1.054, 95% CI (1.010-1.101), *P*=0.02; OR=1.078, 95% CI (1.029-1.129), *P*<0.01]. The results of univariate analysis are listed in Table 2.

**Table 2:** Univariate logistic regression analysis for predicting carotid artery stenosis.

	OR	95% CI	*P*
Age (years)	1.083	1.030-1.138	0.02
Sex (male)	1.440	0.621-3.341	0.40
BMI (kg/m^2^)	0.977	0.892-1.069	0.60
Smoking	0.935	0.455-1.920	0.85
HT	0.877	0.431-1.784	0.72
DM	1.230	0.593-2.551	0.58
COPD	1.120	0.440-2.849	0.81
PAD	0.450	0.158-1.282	0.14
EF (%)	0.980	0.945-1.017	0.29
eGFR (mL/min/1.73m^2^)	1.001	0.984-1.019	0.91
LDL	1.002	0.992-1.011	0.71
CRP	0.994	0.906-1.091	0.91
SYNTAX I	0.988	0.964-1.014	0.37
SYNTAX II PCI	1.050	1.008-1.095	0.02
SYNTAX II CABG	1.078	1.029-1.129	<0.01

BMI=body mass index; CABG=coronary artery bypass grafting; COPD=chronic obstructive pulmonary disease; CRP=C-reactive protein; DM=diabetes mellitus; EF=ejection fraction; eGFR=estimated glomerular filtration rate; HT=hypertension ; LDL=low-density lipoprotein; MI=myocardial infarction; PAD=peripheral arterial disease; PCI=percutaneous coronary intervention

**Fig. 1:**
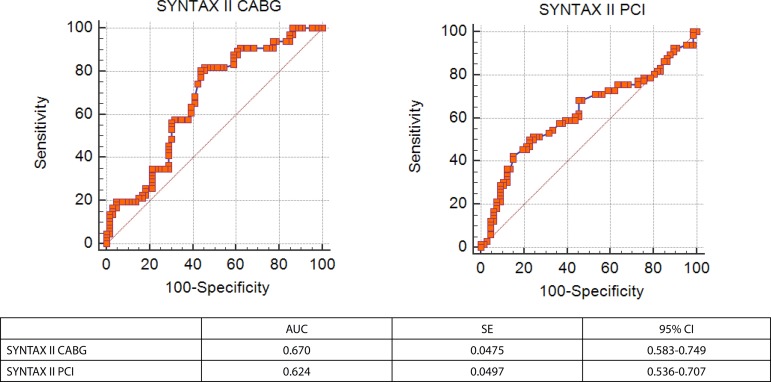
ROC curve analysis of SYNTAX II CABG and PCI for prediction of significant carotid artery stenosis. 95% CI=95% confidence interval; AUC=area under the curve; CABG=coronary artery bypass grafting; PCI=percutaneous coronary intervention; SE=Standard error

**Fig. 2:**
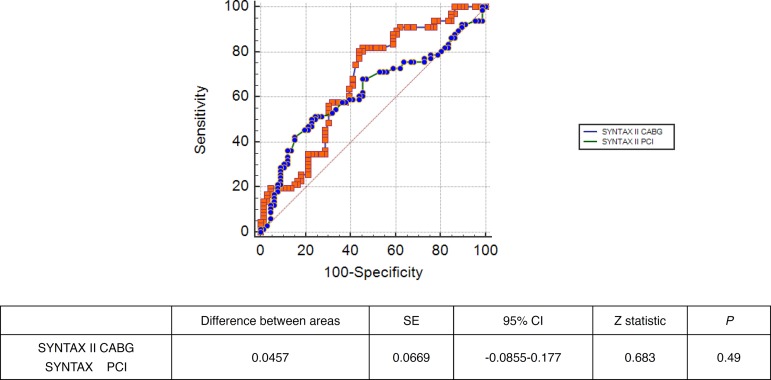
Pairwise comparison of ROC curves of SYNTAX II CABG and PCI for prediction of significant carotid artery stenosis. 95% CI=95% confidence interval; AUC=area under the curve; CABG=coronary artery bypass grafting; PCI=percutaneous coronary intervention; SE=Standard error

ROC curve analysis was performed to predict CAS in the matched population (Figure 1). SS II PCI greater than 33.1 had 68.2% sensitivity and 54.6% specificity [AUC: 0.624, *P*=0.01, 95% CI (0.536-0.707)] whereas SS II CABG greater than 26.1 had 81.8% sensitivity and 54.6% specificity [AUC=0.670, *P*<0.01, 95% CI (0.583-0.749)] to predict CAS. Pairwise comparison of ROC curves revealed similar statistical accuracy of both scoring systems for prediction of CAS (z statistic: 0.683, *P*=0.49) (Figure 2).

## DISCUSSION

Approximately 16% of the patients in our study had significant CAS. The present study demonstrated increased SS II PCI and CABG score in patients with CAS compared to those without CAS by means of propensity score matched analysis. SS II is associated with significant CAS in a population with multivessel disease. SS II CABG had a better diagnostic accuracy, albeit not statistically significant. SS I was not found to be associated with CAS.

Carotid intima-media thickness (CIMT) was shown to be associated with cardiovascular mortality. Several studies demonstrated a close association between CIMT and SS I^[9-11]^. A recent study showed that CIMT correlated with SS II^[12]^. Two previous studies concluded that SS I is not a predictor of CAS^[13,14]^. Compatible with these studies, SS I was not associated with CAS whereas age was an independent predictor of CAS in our study^[14]^. A latter study by the same group of researchers confirmed a relationship between SS II and CAS^[15]^. A recent study by Avci et al.^[13]^ demonstrated the association between SS I and CAS, however, this association was not independent. As argued in various studies, SS is a weighted score taking into account anatomical properties such as tortuosity and calcification, in addition to atherosclerotic lesions. Thus, SS I may be less powerful than Gensini score to predict atherosclerotic burden. Therefore, extent of coronary atherosclerosis may not be thoroughly represented with SS I.

A recent study found CAS as an independent predictor of high SYNTAX score (>32), however, the study population was heterogeneous and comprised patients with single and multivessel disease^[16]^. Up to date, only a single study investigated the relationship between SS II and CAS^[15]^. Baseline characteristics and risk factors of patients in this study were similar to ours. Age was the most important risk factor for CAS in both studies. SS I only reflects anatomical complexity of coronary artery disease. Recently, SS II incorporates clinical factors such as age, sex, and eGFR, in addition to SS I. However, we think high SS II in our study seems to be more related to age and peripheral arterial disease (PAD) rather than other variables in the scoring system, since they are not associated with CAS in the regression analysis. Apart from the previous studies, we used propensity score matching in order to balance patient characteristics. Nevertheless, CAS seems to be more related to age rather than the complexity of coronary atherosclerosis and clinical risk factors in patients with multivessel disease.

### Limitations

The relatively small number of patients and the retrospective nature of the study are major limitations. Since Doppler ultrasound was performed before surgery with the intention to identify critical CAS, CIMT, which may have a further contribution, was not evaluated. Subjects enrolled in our study have multivessel disease and most patients had intermediate to high SS scores. This restriction causes a limitation to generalize the study. Thus, our findings are valid for a restricted patient group.

## CONCLUSION

SS I is not associated with significant carotid artery stenosis. SS II is useful to predict carotid artery stenosis in patients with multivessel coronary artery disease.
